# Preset Hearing Aid Program Selection in Low-Income Communities: A Longitudinal Study

**DOI:** 10.3390/audiolres15050137

**Published:** 2025-10-11

**Authors:** Anné Croucamp, Caitlin Frisby, Vinaya Manchaiah, Tersia de Kock, De Wet Swanepoel

**Affiliations:** 1 Department of Speech-Language Pathology and Audiology, University of Pretoria, Pretoria 0028, Gauteng, South Africa; u21618004@tuks.co.za (A.C.); caitlin.frisby@up.ac.za (C.F.); vinaya.manchaiah@cuanschutz.edu (V.M.); tersia@hearxfoundation.org (T.d.K.); 2 Virtual Hearing Lab, a Collaborative Initiative Between the University of Colorado and the University of Pretoria, Aurora, CO 80045, USA; 3 hearX Foundation, Pretoria 0081, Gauteng, South Africa; 4 Department of Otolaryngology-Head and Neck Surgery, University of Colorado School of Medicine, Aurora, CO 80045, USA; 5 UCHealth Hearing and Balance, University of Colorado Hospital, Aurora, CO 80045, USA; 6 Department of Speech and Hearing, School of Allied Health Sciences, Manipal Academy of Higher Education, Manipal 576104, India

**Keywords:** community health worker, decentralized care, hearing loss, low-income communities, preset hearing aids, program selection

## Abstract

Purpose: Decentralized hearing care models facilitated by community health workers (CHWs) can improve access to care in low-income settings. Preset hearing aids, which offer user-selectable pre-developed amplification programs, may support such models, but little is known about their real-world use and alignment with clinical recommendations. Method: This longitudinal study formed part of a feasibility project implementing the World Health Organization’s (WHO’s) hearing aid service delivery approach in three low-income South African communities. Adults (≥18 years) with confirmed moderate-to-severe bilateral hearing loss were fitted with preset hearing aids by trained CHWs. Devices offered four preset amplification programs. Participant-driven selections were recorded at four distinct time points: fitting and follow-ups at 2 weeks, 2 months, and 6 months post-fitting. Results: In total, 36 participants (mean age = 76 years, SD = 8.9, range 50–96) were fitted with devices. Although over half (right: 52.8%, left: 58.3%) presented with high-frequency loss, only 34% initially selected the corresponding program. Most participants (80.6%, *n* = 29) changed their selections at least once over the six months. Significant associations between hearing loss configuration and selection occurred at some time points only in the left ear, but agreement with clinically recommended programs declined from 42% at baseline to 28% at six months. Conclusions: CHW-facilitated hearing aid delivery supports user autonomy in low-resource settings. However, frequent changes and divergence from clinically recommended programs suggest reliance on user-driven trial-and-error adjustments rather than audiometric fit, which may limit long-term benefit. Ongoing, person-centred guidance is needed for the effective use of preset hearing aids.

## 1. Introduction

Hearing loss is one of the most prevalent sensory impairments worldwide, expected to affect one in four people by 2050 [1]. Low- and middle-income (LMI) settings bear a disproportionate burden [2,3], with an estimated 80% of people with disabling hearing loss residing in these countries [1]. The higher incidences in these settings are likely linked to communicable diseases (e.g., maternal rubella, otitis media), ototoxic medications, and limited access to hearing care services [4,5,6,7]. Barriers include workforce shortages, with fewer than one audiologist per million people in 76% of LMI settings, centralized service models that require expensive travel, the high cost of devices, limited awareness of hearing loss and available treatment, and low health literacy and access to specialized services that may not be covered by private insurance or the government [8,9,10,11,12].

Without timely intervention, hearing loss can profoundly impair communication, physical and mental health, quality of life, and is even associated with cognitive decline and depression [1,3,13]. Although hearing aids are the most common treatment for hearing loss, their use remains low [14,15,16]. In LMI settings, fewer than 12% of people’s hearing needs are being met, with hearing aid adoption rate as low as 2% across Africa [2]. In low-income South African communities, 56% of retirees reported hearing difficulties, but access to hearing aids and affordable care was minimal [17].

Recent World Health Organization (WHO) guidelines promote innovative models, including task-sharing among community health workers (CHWs) and the adoption of digital tools, to strengthen hearing care delivery in resource-limited settings [18,19]. Task-sharing aims to address the shortage of hearing healthcare professionals by enabling trained CHWs to serve as the primary point of care by raising awareness, providing counselling, facilitating device management, and delivering basic hearing care [8,10,19]. Digital innovations can support task-sharing by allowing CHWs to facilitate automated hearing testing, quality controls, remote support, and data management [20,21,22]. Studies in LMI contexts have already demonstrated that such models are feasible, with CHWs successfully trained to provide hearing screenings, fittings, and follow-up support [23,24,25,26]. The WHO guidelines for these settings recommend low-cost, high-quality preset hearing aids to improve access in CHW-facilitated models [19].

Preset hearing aids are pre-programmed for common hearing loss configurations and allow only basic adjustments, such as volume control, without requiring clinical customization using a person’s hearing sensitivity or the use of software [27,28]. While only formally permitted for direct purchase without clinical consultation in the United States as one of the Food and Drug Administration’s (FDA) over-the-counter (OTC) hearing aid categories (preset, self-fitting, and software as a medical device), such models are increasingly relevant for decentralized service delivery in LMI settings where audiological infrastructure is limited [27,29,30,31]. The features and functionalities, as well as the audio performance of the present hearing devices, vary substantially within and between countries [32]. Due to a lack of standard terminologies adopted, often these devices may be referred to as personal sound amplification products (PSAPs), hearing amplifiers, and sometimes preset hearing aids, depending on the context and type of device.

Some early research evidence suggests that preset hearing devices can produce self-reported outcomes similar to traditional audiogram-based fittings in terms of listening effort, sound quality, speech recognition, and user satisfaction, particularly for adults with mild to moderate hearing loss [33,34,35]. Venkitakrishnan, Urbanski and Wu [35] directly compared user preferences across laboratory-developed preset, self-fitting, and audiogram-based hearing aids, reporting that 54% of participants preferred the preset devices. However, limitations persist, including their suitability for more complex hearing loss configurations, such as rising, notched, or steeply sloping losses, and the reliance on users to self-select appropriate programs due to the devices’ lack of personalized customization options [33,35]. A recent randomized controlled trial by Wu, et al. [36] found that while advanced, audiologist-fitted models demonstrated superior performance in global benefit scores and user satisfaction, as measured through the ecological momentary assessment Glasgow Hearing Aid Benefit Profile (EMA-GHAB), users of simpler, low-tech preset hearing aids still reported meaningful benefit after six weeks [36]. Importantly, the study also found no significant differences in outcomes between low-end and high-end hearing aids. More recently, research on instant-fit devices has shown that open completely-in-the-canal styles could also offer a comfortable, discreet, and high-performance option, addressing the aesthetic concerns that often deter hearing aid use [37]. These findings highlight that although high-tech prescription-based fittings facilitated by professionals may yield better outcomes, low-tech, preset hearing devices remain a viable and effective alternative, particularly in settings where audiologist-led care is inaccessible. However, access to affordable devices alone is not sufficient to close hearing care gaps [38]. Addressing this requires evidence on the usability, feasibility, and efficacy of currently available preset hearing aids, which remains scarce in community-based settings [31].

Despite growing evidence and emerging innovations, user-initiated program selection of preset programs in low-income settings remains relatively unstudied. Critical questions remain about real-world use and longitudinal outcomes of preset-based OTC hearing aids [31,39]. It is unclear whether users’ programs align with their hearing needs, how user preferences evolve over a period of six months, and whether such changes are associated with improved self-reported outcomes. Therefore, this study explored how adults in low-income settings, fitted with hearing aids by CHWs, selected and adjusted programs over time, providing insight into user behaviour and preferences in community-based hearing care.

## 2. Materials and Methods

### 2.1. Study Design

This retrospective, longitudinal study was embedded in a larger feasibility project evaluating the implementation of the WHO hearing aid service delivery approach for LMI settings [19]. Institutional review board clearance was granted by the Research Ethics Committee, Faculty of Humanities, University of Pretoria (HUM038/0425).

The study assessed how user preferences evolve by comparing initial program selections with those recorded at various follow-ups, including two weeks, two months, and six months post hearing aid fitting. Additionally, the study explored whether there was a correspondence between individuals’ audiometric profiles, including the degree and configuration of their hearing loss, and their selected preset hearing aid programs.

### 2.2. Setting

The feasibility study was conducted in three low-income communities in South Africa in partnership with the hearX Foundation, a local NGO training CHWs for community-based service delivery. The communities included Atteridgeville (Gauteng), Khayelitsha, and Mbekweni (Western Cape). These communities present with barriers to healthcare that include limited resources, professionals, and low health literacy. Khayelitsha is one of the most densely populated townships in Cape Town, residing almost 400,000 people more than a decade ago [40], whereas Mbekweni only had approximately 30,875 residents at that time [41]. Common challenges across these settings include high population density, unemployment and poverty rates, poor infrastructure, crime, and restricted access to healthcare services [42,43,44,45].

### 2.3. Participants

#### 2.3.1. CHW Recruitment and Training

CHWs, employed by the partner NGO, were purposively recruited and trained to facilitate the service delivery. Two audiologists conducted the in-person training covering the following modules: basic introductions to hearing, preparation for assessment, introduction to otoscopy and assessment, special considerations when testing difficult-to-test patients, completing the assessment process, basic troubleshooting and equipment maintenance, introduction to hearing aid fitting, and hearing aid follow-up and troubleshooting.

CHWs underwent a practical component whereby they needed to conduct the full service-delivery model on individuals under the supervision of the audiologist. Competency was assessed through an audiologist evaluation. The evaluation included a five-point rating scale on attendance, familiarity with tools, interpersonal skills, and overall motivation. Ratings were ranged from “Not Ready” to “Fully Comfortable.”

All CHWs obtained a “fully comfortable” score across all five items, except one CHW, who indicated a “semi-comfortable” score on familiarity with assessment and hearing devices, indicating that the CHW was mostly independent but occasionally may have needed to self-reorient or pause to remember the next steps while still being cleared to work without in-person supervision.

#### 2.3.2. Participant Recruitment

Participants’ inclusion criteria to be considered for testing: 18 years and older; residing in the LMI communities; self-reported suspected hearing loss. Participants were recruited through a snowball sampling strategy using elderly clubs and community leaders. In order to be eligible for a hearing aid fitting, participants needed to present with an air conduction pure tone average (at 0.5, 1, 2, and 4 kHz) between 35 and 79 dB HL bilaterally, even if asymmetrical, as recommended by the WHO hearing aid service delivery approach for LMI settings [19].

### 2.4. Material and Apparatus

The study employed three main tools: a mobile device-operated (1) video-otoscope and (2) audiometer, and (3) preset hearing aids. Video-otoscopy was conducted using the hearScope™ device (hearX Group, Pretoria, South Africa), which incorporated an artificial intelligence-based classification for common ear pathologies (beta version). Categories included: (i) Normal, representing a clear and healthy outer ear and tympanic membrane; (ii) Abnormal, indicating any structural anomalies or disorder of the outer/middle ear (iii) Cerumen impaction (iv) Unable to determine, which represents all otoscopic results that could not be classified due to factors such as low-resolution imaging of the ear canal, lack of training data, and complex or atypical cases [46]. This device was operated using either a Samsung SM-T515 tablet or SM-A04 smartphone (Samsung, Pretoria, South Africa). On the same device, audiometric testing was performed using the hearTest™ application (hearX Group, Pretoria, South Africa), measuring air conduction thresholds at 0.5, 1, 2, and 4 kHz. Testing utilized Sennheiser HD280 Pro headphones (Sennheiser, Pretoria, South Africa), adhering to calibration and test condition standards (ISO 389-1:2017; ISO 389-9:2014) [47,48], with thresholds obtained via the Shortened Ascending method (ISO 8253-1:2010) [49]. Participants were fitted with Go Ultra™ preset based OTC hearing aids (Go Hearing™), which are rechargeable, behind-the-ear devices programmed with four preset programs: (1) general-purpose program with a balanced, frequency-shaped response prioritizing speech audibility, (2) high-frequency program designed for hearing profiles with loss in the high frequencies, (3) low-frequency program for individuals with hearing loss in the lower frequencies, and (4) program that applies a broadband gain across low- to high-frequencies relative to Program 1, preserving spectral shape while raising overall loudness (Appendix A). Program selection could be altered using a control switch located on the hearing aid. At the time of the study, these devices were commercially available only in the United States, retailing at approximately $300 per pair through direct-to-consumer (DTC) channels.

### 2.5. Procedure

This study focused on the hearing aid fitting and follow-up phase of a broader community-based hearing care initiative, implemented in accordance with the WHO hearing aid service delivery approach for LMI settings [19]. All procedures were facilitated by the CHWs and are detailed below.

#### 2.5.1. Screening and Assessment

Over a 16-month period, CHWs screened 166 adults. Demographic and hearing health information (red flags such as ear pain, bleeding in the ear, infections, sudden or unilateral hearing loss experienced within the previous 3 months) were collected, followed by otoscopic examinations. Individuals presenting with cerumen impaction or other abnormalities were referred to the local healthcare system and reassessed following resolution. Subsequently, audiometric testing was conducted. Participants identified with a mild degree of hearing loss received counselling and were advised to undergo annual monitoring. Those with moderate, moderately severe, or severe hearing loss bilaterally met the eligibility criteria for hearing aid fitting. Participants with profound hearing loss were counselled and referred to a hearing specialist as they required stronger amplification.

#### 2.5.2. Hearing Aid Fitting and Follow-Up

Participants who met the inclusion criteria (pure tone average between 35 and 79 dB HL bilaterally) were offered a bilateral hearing aid fitting. In a single session, each participant trialed the four preset amplification programs through in situ listening comparisons facilitated by a CHW, using conversational speech to guide the selection. Program selection was participant-driven, following CHW-facilitated in situ listening trials on each of the programs. Participants were informed of their ability to adjust programs at any time.

Participants received a four-week mHealth support program delivered via WhatsApp or SMS, offering guidance on hearing aid use, maintenance, and basic troubleshooting. Complementing this digital support, the CHWs provided in-person orientation on device-handling, care, and troubleshooting procedures. Devices were given to participants to retain after study completion.

Program selections were recorded exclusively at four discrete time points: initial fitting, and at follow-up visits occurring two weeks, two months, and six months post- hearing aid fitting. CHWs documented selections using a paper-based system. No additional information was recorded regarding the timing or process of the selection.

#### 2.5.3. Clinically Recommended Program

Two audiologists (AC, CF) retrospectively discussed and assigned clinically recommended programs based on each participant’s audiometric profile, specifically the configuration of their hearing loss (Table 1). Recommendations followed predefined mapping rules, developed collaboratively by researchers and clinical audiologists, which matched common hearing loss configurations (e.g., flat, sloping, rising) to the preset program most closely aligned with typical amplification needs for that configuration. These recommendations were not disclosed to participants or CHWs.

### 2.6. Data Analysis

Raw data were recorded in Microsoft Excel and analysed using SPSS V.30 (Statistical Package for the Social Sciences). The four-frequency pure tone average (FPTA) (at 0.5, 1, 2, and 4 kHz) was utilized to categorize hearing loss degree as mild (20–34 dB HL), moderate (35–49 dB HL), moderately severe (50–64 dB HL), severe (65–79 dB HL), or profound (80–94 dB HL) according to WHO guidelines [1]. Configurations were defined as flat (<20 dB HL difference across frequencies), high-frequency (>20 dB HL difference, greatest loss in the higher frequencies), or low-frequency loss (>20 dB HL difference, greatest loss in the lower frequencies).

Descriptive statistics (means, standard deviations, frequencies, and percentages) were computed to summarize participant demographics, hearing loss characteristics, and hearing aid program selections.

Agreement between clinically recommended and participant-selected programs across time points was assessed using binary coding (match vs. mismatch), with Cohen’s Kappa to quantify agreement. Agreement between clinically recommended and participant-selected programs was quantified with Cohen’s κ (with 95% CIs) and overall percent agreement. Interpretation followed Landis and Koch [50] categories: <0.00 poor; 0.00–0.20 slight; 0.21–0.40 fair; 0.41–0.60 moderate; 0.61–0.80 substantial; 0.81–1.00 almost perfect.

Missing follow-up data were handled using listwise deletion. Follow-up attendance was: fitting *n* = 35, 2 weeks *n* = 30, 2 months *n* = 32, and 6 months *n* = 29. Analyses of changes between consecutive visits were restricted 8–9 to participants observed at both visits (complete-case, as-observed). Given the small sample, listwise deletion may bias estimates if data are not missing completely at random; therefore, we report the denominators for each analysis and interpret exploratory *p*-values cautiously. Where feasible, we provide an available-case sensitivity check to verify that effect direction/magnitude is consistent.

#### Program Selection and Audiometric Configurations

To assess associations between audiometric profiles (configuration and degree) and program selections, Chi-square tests with Monte Carlo simulations (to account for small cell sizes) were used. Cochran’s Q and McNemar tests evaluated changes in program selection over time. Visualizations (bar charts, heatmaps) illustrated selection trends and configuration-based differences. Given the study’s exploratory aims and small sample, we retained α = 0.05 (two-tailed) without applying Bonferroni-type family-wise error corrections to avoid inflating Type II error. We report exact *p*-values and emphasize effect sizes with 95% confidence intervals (Cramér’s V for χ^2^; Cohen’s κ with 95% CIs), along with transparent disclosure of the number and nature of tests performed. Results are interpreted cautiously, prioritizing the magnitude and precision of effects over dichotomous significance.

The sample sizes presented in the figures reflect the total number of participants who attended each respective session (e.g., fitting, 2-week follow-up, 2-month follow-up, 6-month follow-up). In contrast, the descriptive statistics on changes in hearing aid program selection include participants who attended both consecutive sessions, resulting in lower *n* values.

## 3. Results

### 3.1. Recruitment and Demographics

**Figure 1:**
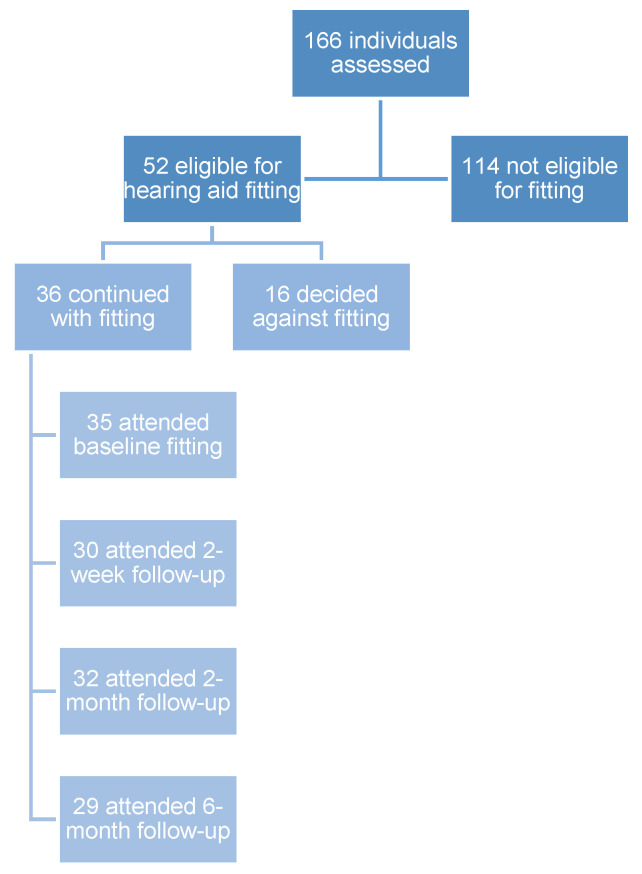
Consort diagram illustrating participant recruitment and follow-up attendance.

Of the 166 individuals assessed, 36 participants were fitted with hearing aids (Figure 1). 16 eligible individuals declined the fitting (30.8%), citing reasons such as lack of interest in hearing aids or no perceived hearing difficulty. The average age of the fitted participants was 76 years (SD = 8.9; range 50–96), with 63.9% (*n* = 23) female and 36.1% (*n* = 13) male participants. Figure 1 illustrates participant recruitment and follow-up attendance numbers. Attendance and analysis denominators were: fitting *n* = 35, 2 weeks *n* = 30, 2 months *n* = 32, and 6 months *n* = 29. For any consecutive-visit comparison, the *n* reflects only participants present at both visits; these denominators are shown in the table/figure footnotes. Participant data was not recorded if they did not attend the scheduled in-person visits, did not bring their hearing aids, or completed a phone interview where CHWs could not manually check program selections.

### 3.2. Hearing Characteristics

Most participants presented with moderate hearing loss in the better ear (55.6%, *n* = 20) and a high-frequency configuration (Right: 52.8%, *n* = 19; Left: 58.3%, *n* = 21). Table 2 summarizes the distribution of the degree and configuration of hearing loss across ears.

### 3.3. Hearing Aid Program Selection

**Figure 2:**
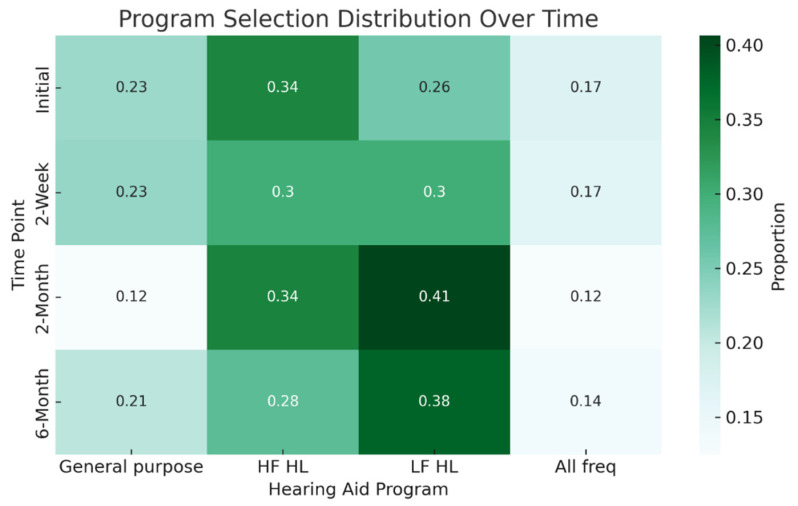
Distribution of selected hearing aid programs at the initial fitting (*n* = 35) and during follow-up sessions at 2 weeks (*n* = 30), 2 months (*n* = 32), and 6 months (*n* = 29). (General Purpose—program 1; High-Frequency Hearing Loss, HF HL—program 2; Low-Frequency Hearing Loss, LF HL—program 3; All Frequencies—program 4).

Figure 2 visually illustrates the distribution of the participants’ program selection at each time point. All participants used the same program for both ears. At the initial fitting, the High-Frequency Amplification program (Program 2) was the most frequently selected (34%, *n* = 12). This was followed by the Low-Frequency Amplification program (Program 3, 26%, *n* = 9), the General-Purpose program (Program 1, 23%, *n* = 8), and All-Frequencies Amplification program (Program 4, 17%, *n* = 6).

Changes in hearing aid program selection varied across follow-up points. At the 2-week follow-up, 13.8% (*n* = 4) of the 29 returning participants had changed their program selection since the initial fitting. Among the 28 participants who attended both the 2-month and 6-month follow-ups, 39.3% (*n* = 11) changed their selection between baseline and 2 months, and 53.6% (*n* = 15) made changes between 2 and 6 months.

Overall, 26.9% of participants (*n* = 7) made no changes to their selected program, almost half (42.3%, *n* = 11) made a single change, while 26.9% (*n* = 7) altered their selection twice. Only one participant (3.8%) changed their selected program at every follow-up session.

Most of those who made no changes (57.1%; *n* = 4) selected the Low-Frequency amplification program (Program 3) at baseline (28.6% Program 4 and 14.3% Program 2). There was no significant age difference between participants who changed their hearing aid program during follow-up and those who did not (*p* = 0.57, Independent samples *t*-test).

### 3.4. Hearing Loss Degree and Program Selection

No significant associations were found between hearing loss degree and program selection at any time point (*p* > 0.05).

### 3.5. Configuration and Program Selection

**Figure 3:**
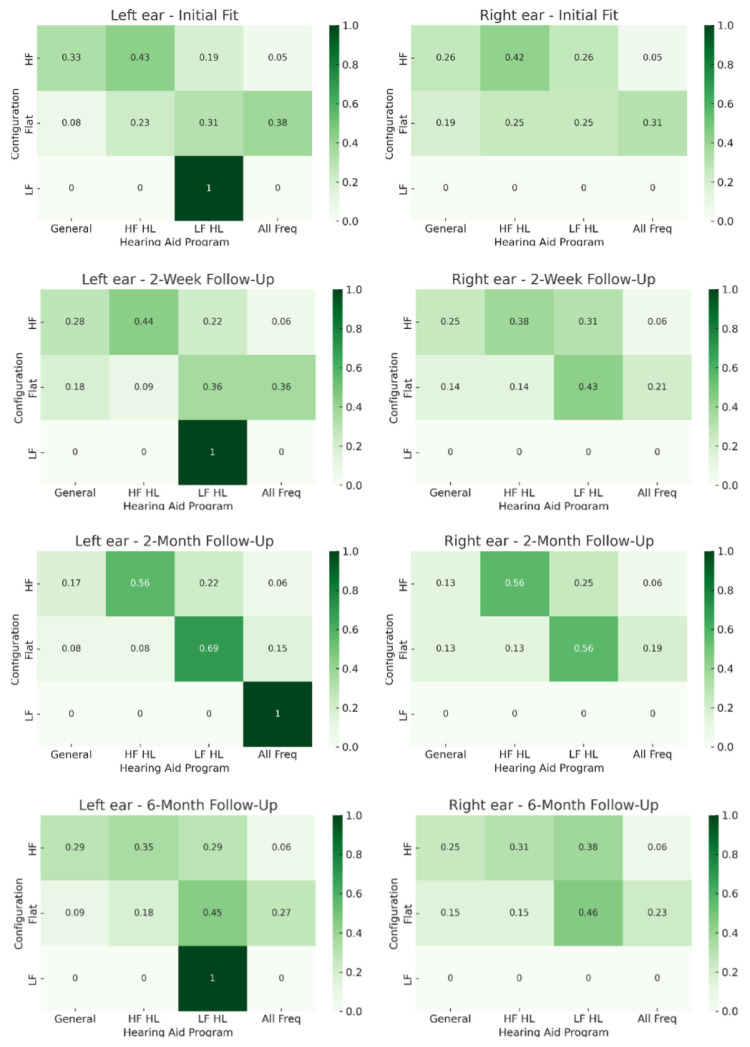
Distribution of selected hearing-aid programs by hearing-loss configuration across visits (rows) and ear (columns). (Left column: left ear; right column: right ear. Visits and sample sizes: initial fitting (*n* = 35), 2 weeks (*n* = 30), 2 months (*n* = 32), and 6 months (*n* = 29). Program codes: 1 = General Purpose; 2 = High-Frequency Hearing Loss (HF HL); 3 = Low-Frequency Hearing Loss (LF HL); 4 = All Frequencies).

Program selection varied by hearing loss configuration as shown in the combined-ear heatmaps (Figure 3). In participants with high-frequency hearing loss, the High-Frequency Amplification program (Program 2) was most frequently chosen (left ear: 43%; right ear: 42%). Over time, those with flat configurations tended toward selection of the Low-Frequency Amplification program (Program 3; left ear: initially 31% to 45% at 6 months, right ear: initially 25% to 46% at 6 months).

Statistically significant associations between hearing loss configuration and program selection were observed in the left ear baseline (*p* = 0.032) and at two months (*p* = 0.003). No significant associations were, however, found for the right ear at any of the time points.

To further explore the relationship between hearing loss configuration and program selection, analyses were conducted using the configuration of the best ear (based on 4FPTA). At the initial fitting, the overall association between hearing loss configuration and program selection was not statistically significant (*p* = 0.091). However, a significant linear-by-linear association suggested the presence of a monotonic trend across configuration and selection categories (*p* = 0.026), indicating selections may have been influenced by their configuration, even in the absence of a strong overall association. A similar pattern was seen at the two-week follow-up, where the overall association remained non-significant (*p* = 0.067), but the linear trend across configurations and selected programs persisted (*p* = 0.045). By the two-month follow-up, both the overall association (*p* = 0.023) and the linear-by-linear trend reached statistical significance (*p* = 0.013), suggesting a stronger and more consistent alignment between users’ program selections and their audiometric configurations. However, by the six-month follow-up, this alignment weakened, with no significant association (*p* = 0.253) and a linear trend that approached but did not reach significance (*p* = 0.052), indicating a possible decline in alignment over time.

### 3.6. Participant Selection vs. Clinically Recommended Program

The proportion of participants whose selected programs matched clinical recommendations did not differ significantly across the four follow-up sessions in either the left ear (*p* = 0.249, Cochran’s Q test) or right ear (*p* = 0.129, Cochran’s Q test). Similarly, pairwise comparisons between consecutive time points showed no significant changes in alignment (*p* > 0.05, McNemar test). Agreement levels between participant selection and clinically recommended programs, measured using Cohen’s Kappa, were generally low (κ categories per Landis and Koch [50]: <0.00 poor; 0.00–0.20 slight; 0.21–0.40 fair; 0.41–0.60 moderate; 0.61–0.80 substantial; 0.81–1.00 almost perfect. κ reported with 95% CIs and percent agreement). For the left ear, agreement was fair at initial fitting (κ = 0.210, *p* < 0.001, 95% CI [0.03, 0.39]) and at two weeks (κ = 0.243, *p* < 0.001, 95% CI [0.07, 0.42]), but declined to slight agreement at both two months (κ = 0.152, *p* < 0.001, 95% CI [0.01, 0.30]) and six months (κ = 0.152, *p* < 0.001, 95% CI [−0.01, 0.32]). The right ear showed slight agreement throughout: lowest at the initial fitting (κ = 0.145, *p* = 0.068, 95% CI [0.01, 0.30]) and two weeks (κ = 0.125, *p* = 0.117, 95% CI [−0.03, 0.28]), peaking modestly at two months (κ = 0.184, *p* = 0.014, 95% CI [0.04, 0.33]), and decreasing again by six months (κ = 0.100, *p* = 0.149, 95% CI [−0.04, 0.24]).

**Figure 4:**
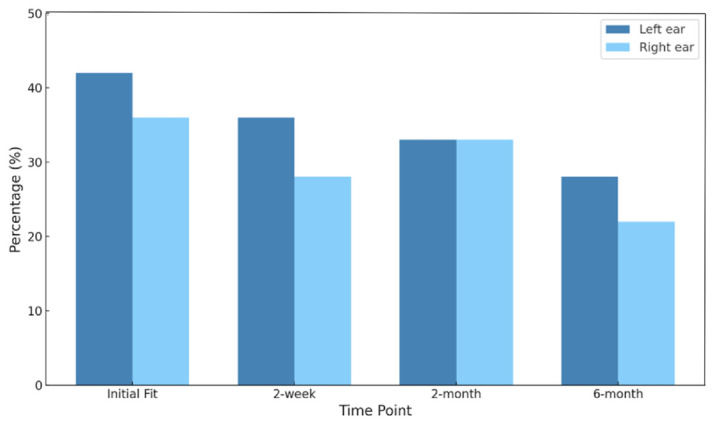
Distribution of participants selecting the clinically recommended program at the initial fitting (*n* = 35), 2-week (*n* = 30), 2-month (*n* = 32), and 6-month (*n* = 29) follow-ups.

As shown in Figure 4, the proportion of participants selecting the clinically recommended program declined gradually across the follow-up time points. For the left ear, 41.7% (*n* = 15) initially selected the clinically recommended program, but only 27.8% (*n* = 10) had the clinically recommended program selected by the 6-month follow-up. A similar trend was observed for the right ear, decreasing from 36.1% (*n* = 13) to 22.2% (*n* = 8).

## 4. Discussion

This study examined self-selected preset hearing aid program use over six months in adults from low-income communities and evaluated the extent to which selections aligned with audiometric profiles and clinical recommendations. Most participants (80.6%) changed their selection at least once, reflecting dynamic usage patterns. While the High-Frequency Amplification program (Program 2) was most frequently selected at the initial fitting, corresponding to the predominance of high-frequency hearing loss, the Low-Frequency Amplification (Program 3) became most common by six months.

Only 19.4% of participants maintained the same program throughout, suggesting either a strong initial match or influences such as user confidence, proficiency with digital devices, education, or limited willingness to experiment [51]. This variability in program use likely reflects an adaptation period as users experimented and adjusted selections to their preferences. This aligns with evidence that acclimatization to hearing aids typically occurs within the first 12 weeks of use [52,53] and that gain preferences also evolve with experience [54,55]. These findings underscore the multifactorial nature of hearing aid use and the need for guided trial periods during hearing aid adoption. Supporting this, Urbanski, Hernandez, Oleson and Wu [33] found that users could independently complete the preset selection process in 15 min with outcomes comparable to clinically fitted devices. Nonetheless, variability in selections and performance remained.

Degree of hearing loss was not significantly associated with program selection, suggesting that degree alone may not reliably predict user preferences. Users may have compensated for suboptimal gain by adjusting volume controls, consistent with Perry, Nelson and Van Tasell [54], who found greater losses led to higher self-selected gain, though variability in preferences may obscure associations. Additionally, recent findings indicate that many new hearing aid users prefer slightly under-amplified fittings for perceived comfort, even when these deviate from prescriptive targets that offer greater clarity [56]. This aligns with recent evidence that user-selected gain below prescription targets can still result in positive outcomes and stable use over time [57]. These findings underscore the influence of subjective factors, such as individual tolerance for high-frequency amplification and the personal trade-off between comfort and clarity, on user preferences, highlighting the need to look beyond audiometric thresholds when making fitting decisions.

Hearing loss configuration showed stronger, though inconsistent, associations with program selection at two months—particularly in the left and better-hearing ear—suggesting user choices may initially align with audiometric needs. This is consistent with adaptation models [54] and speech perception studies showing listeners emphasize frequencies where hearing is better [58,59]. By six months, however, this alignment weakened, as Shen, Yun and Liu [58] found that once speech audibility is restored, reliance on preserved frequency regions declines.

Participant-selected programs increasingly diverged from clinically recommended choices over time as Cohen’s Kappa agreement levels remained slight to fair, potentially driven by users prioritizing comfort or perceived benefit. This is supported by prior work showing that users often prefer self-adjusted over prescriptive settings [60,61]. 

Findings illustrate that users can manage preset hearing aids with a degree of independence, supporting the feasibility of community-based hearing care facilitated by trained CHWs. However, the decline in alignment with clinically recommended programs and hearing loss configuration highlights the need for sustained, person-centred support. Prior studies have shown that such support, whether delivered through CHWs, remote monitoring, or mHealth tools, can support satisfaction and optimize device use [26,62]. At the same time, it remains uncertain whether user-selected programs, chosen for comfort, listening preference, or situational fit, improve real-world adherence, daily wear time, and patient-reported outcomes compared with clinician-set programs. While audiologist-led fittings typically result in better self-reported and behavioural outcomes [36,63], hybrid models that combine user autonomy with structured support (e.g., via CHWs or apps) may offer scalable solutions for underserved communities [29]. Rigorous comparisons of program-selection strategies are required to determine which approach maximizes long-term use and outcomes.

This study has several limitations. Participants received no guidance on program selection, limiting insight into how recommendations might shape behaviour. Reported choices were recorded only at follow-up visits; without data logging, interim switching could not be captured. Reasons and contextual factors behind program choices were also not explored. The relatively small sample reduces statistical power and generalizability, and missing follow-up data may have influenced trends. Analyses focused on hearing loss configuration and degree, excluding other influences such as cognition or psychoacoustic preferences. Outcomes relied on self-reported selections rather than performance measures, limiting conclusions about functional benefit. Finally, we did not apply Bonferroni-type adjustments for multiple comparisons to avoid excess Type II error in this small exploratory dataset; we therefore report exact *p*-values and effect sizes and interpret findings cautiously. Future research should include larger, more diverse samples and qualitative methods to better capture decision-making.

## 5. Conclusions

This study explored how adults in low-income communities select and change preset hearing aid programs over time, offering insight into user behaviour across a six-month period. While preset hearing aids allow CHW-facilitated fittings in low-income communities, program selections shifted over time and often diverged from clinical recommendations. These patterns plausibly suggest that users may have prioritized comfort and real-world preferences, while also demonstrating their potential to take an active role in managing device use. To optimize outcomes, ongoing guided support by professionals or trained CHWs should be available in community-based hearing care whether in-person or using telehealth.

## Figures and Tables

**Table 1:** Distribution of patient selected and clinically recommended hearing aid programs for the better-hearing ear (*n* = 36).

	General Purpose % (*n*)	High Frequency HL % (*n*)	Low Frequency HL % (*n*)	All Frequencies % (*n*)
Clinical recommendation	-	58.3 (21)	-	41.7 (15)
Participant selection				
Initial	22.9 (8)	34.3 (12)	25.7 (9)	17.1 (6)
2-week	23.3 (7)	30.0 (9)	30.0(9)	16.7 (5)
2-month	12.5 (4)	34.1 (11)	40.6 (13)	12.5 (4)
6-month	20.7 (6)	27.6 (8)	37.9 (11)	13.8 (4)

**Table 2:** Degree (4 frequency pure tone average) and configuration of hearing loss in the left and right ear (*n* = 36).

	Right Ears % (*n*)	Left Ears % (*n*)
Degree (4FPTA)		
Moderate (35–49 dB HL)	55.6 (20)	55.6 (20)
Moderately severe (50–64 dB HL)	36.1 (13)	33.3 (12)
Severe (65–79 dB HL)	8.3 (3)	11.1 (4)
Configuration *		
High-frequency loss	52.8 (19)	58.3 (21)
Flat loss	47.2 (17)	38.9 (14)
Low-frequency loss	0 (0)	2.8 (1)

* flat (<20 dB HL difference across frequencies), high-frequency (>20 dB HL difference, greatest loss in the higher frequencies), low-frequency loss (>20 dB HL difference, greatest loss in the lower frequencies).

**Figure A1:**
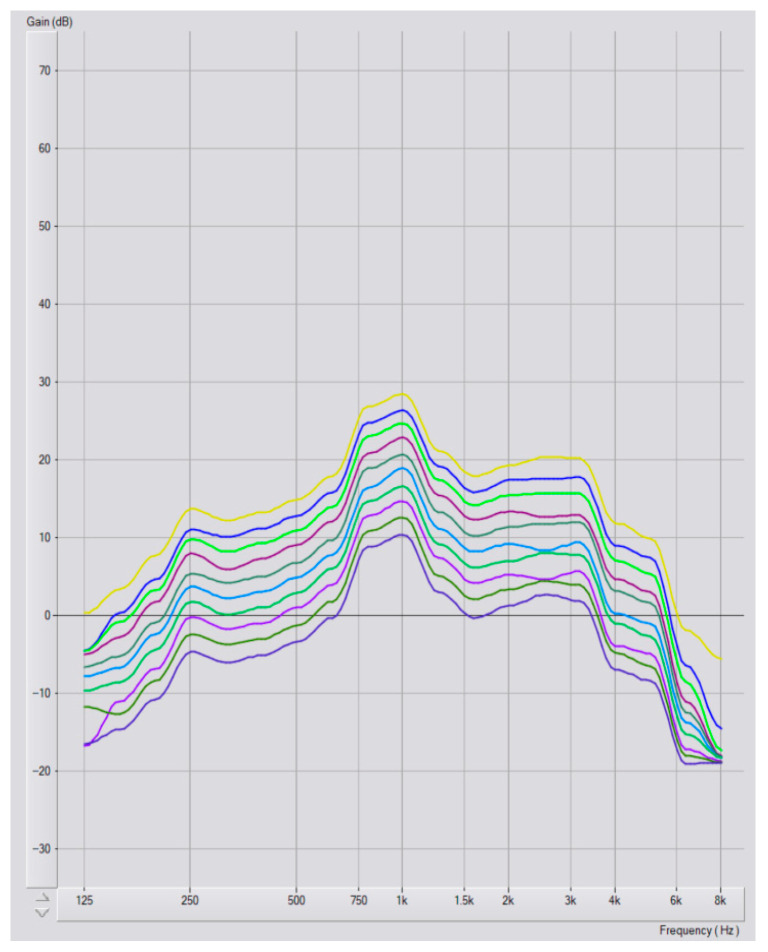
Program 1—General Purpose.

**Figure A2:**
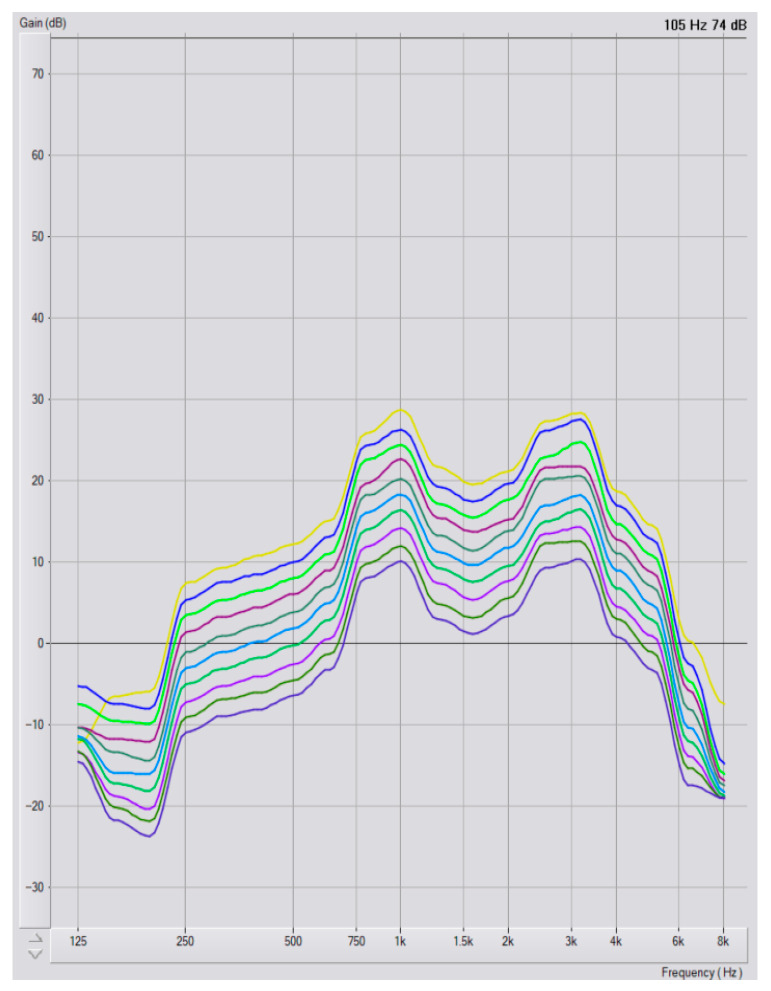
Program 2—High-frequency loss.

**Figure A3:**
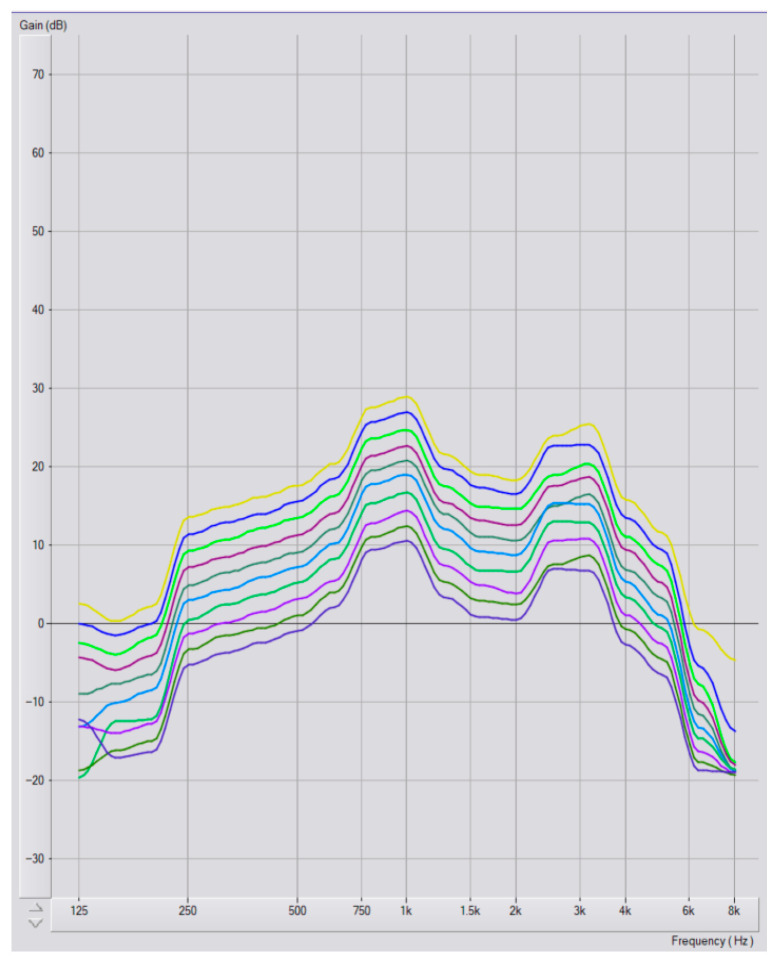
Program 3—Low-frequency loss.

**Figure A4:**
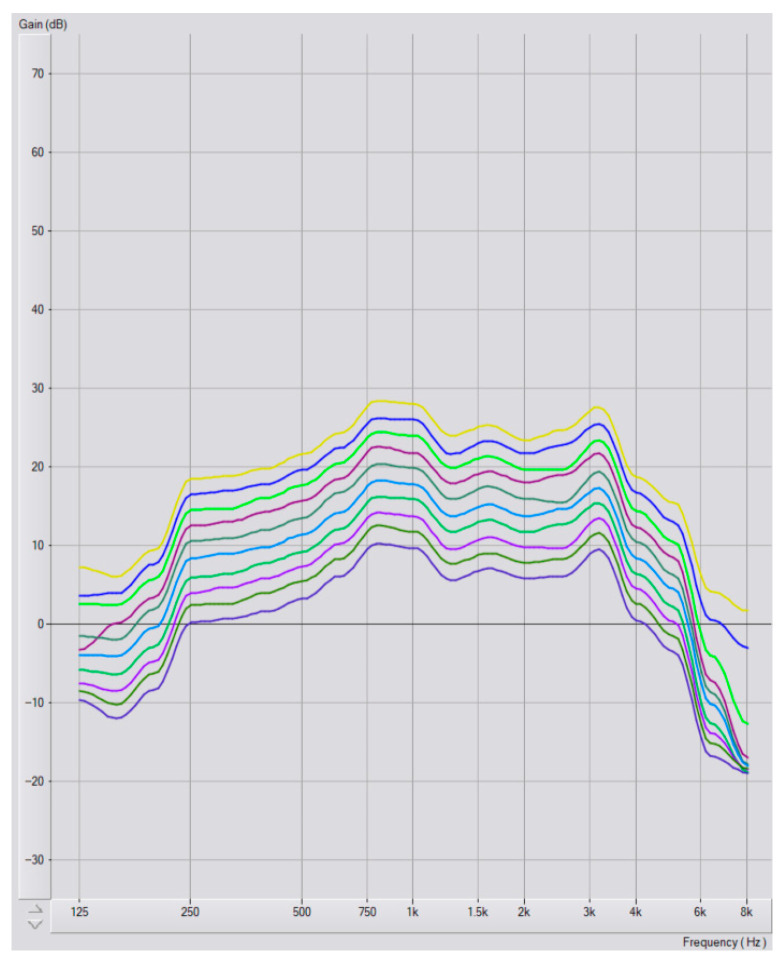
Program 4—All frequencies amplified.

## Data Availability

The datasets used and/or analyzed during the current study are available from the corresponding author upon reasonable request.

## Abbreviations

The following abbreviations are used in this manuscript:

4FPTA	Four-Frequency Pure-Tone Average
CHW	Community Health Worker
EMA-GHAB	Ecological Momentary Assessment Glasgow Hearing Aid Benefit Profile
LMI	Low- and Middle-Income
mHealth	Mobile Health
NGO	Non-Governmental Organization
OTC	Over-the-Counter
PSAP	Personal Sound Amplification Product
PTA	Pure-Tone-Average
US	United States
WHO	World Health Organization

## Appendix A

The Go Ultra Hearing Aid was tested for each program to determine the frequency output at every volume level with the use of a 2cc coupler. The volume increases by approximately 2 dB per volume change increment. The figures below (Figure A1, Figure A2, Figure A3 and Figure A4) represent these outputs across 10 different volume levels for each program (The blue line indicates the minimum volume and the yellow line indicates the maximum volume, with other lines indicating outputs between the maximum and minimum volumes).
